# Cognitive Impairments in Drug-Naive Patients With First-Episode Negative Symptom–Dominant Psychosis

**DOI:** 10.1001/jamanetworkopen.2024.15110

**Published:** 2024-06-06

**Authors:** TianHong Zhang, YanYan Wei, XiaoChen Tang, HuiRu Cui, YeGang Hu, LiHua Xu, HaiChun Liu, ZiXuan Wang, Tao Chen, Qiang Hu, ChunBo Li, JiJun Wang

**Affiliations:** 1 Shanghai Mental Health Center, Shanghai Jiaotong University School of Medicine, Shanghai Engineering Research Center of Intelligent Psychological Evaluation and Intervention, Shanghai Key Laboratory of Psychotic Disorders, Shanghai, PR China; 2 Department of Automation, Shanghai Jiao Tong University, Shanghai, PR China; 3 Shanghai Xinlianxin Psychological Counseling Center, Shanghai, PR China; 4 Big Data Research Lab, University of Waterloo, Waterloo, Ontario, Canada; 5 Labor and Worklife Program, Harvard University, Cambridge, Massachusetts; 6 Department of Psychiatry, ZhenJiang Mental Health Center, Zhenjiang, PR China; 7 Center for Excellence in Brain Science and Intelligence Technology (CEBSIT), Chinese Academy of Science, Shanghai, PR China; 8 Institute of Psychology and Behavioral Science, Shanghai Jiao Tong University, Shanghai, PR China

## Abstract

**Importance:**

Available antipsychotic medications are predominantly used to treat positive symptoms, such as hallucinations and delusions, in patients with first-episode psychosis (FEP). However, treating negative and cognitive symptoms, which are closely related to functional outcomes, remains a challenge.

**Objective:**

To explore the cognitive characteristics of patients with negative symptom–dominant (NSD) psychosis.

**Design, Setting, and Participants:**

This large-scale cross-sectional study of patients with FEP was led by the Shanghai Mental Health Center in China from 2016 to 2021, with participants recruited from 10 psychiatric tertiary hospitals. A comprehensive cognitive assessment was performed among 788 patients with FEP who were drug-naive. Symptom profiles were determined using the Positive and Negative Symptoms Scale (PANSS), and NSD was defined as a PANSS score for negative symptoms higher than that for positive and general symptoms. Positive symptom–dominant (PSD) and general symptom–dominant (GSD) psychosis were defined similarly. Data were analyzed in 2023.

**Exposure:**

Psychotic symptoms were categorized into 3 groups: NSD, PSD, and GSD.

**Main Outcomes and Measures:**

Neurocognitive performance, assessed using the Chinese version of the Measurement and Treatment Research to Improve Cognition in Schizophrenia Consensus Cognitive Battery.

**Results:**

This study included 788 individuals with FEP (median age, 22 [IQR, 17-28] years; 399 men [50.6%]). Patients with NSD exhibited more-pronounced cognitive impairment than did those with PSD or GSD. Specifically, cognitive differences between the NSD and PSD group, as well as between the NSD and GSD group, were most notable in the processing speed and attention domains (Trail Making [*F* = 4.410; *P* = .01], Symbol Coding [*F* = 4.957; *P* = .007], Verbal Learning [*F* = 3.198; *P* = .04], and Continuous Performance [*F* = 3.057; *P* = .05]). Patients with PSD and GSD showed no significant cognitive differences. Cognitive impairment was positively associated with the severity of negative symptoms. Most of the cognitive function tests used were able to differentiate patients with NSD from those with PSD and GSD, with significant differences observed across a range of tests, from Brief Visuospatial Memory Test–Revised (*χ^2^* = 3.968; *P* = .05) to Brief Assessment of Cognition in Schizophrenia symbol coding (*χ^2^* = 9.765; *P* = .002).

**Conclusions and Relevance:**

The findings of this cross-sectional study of patients with FEP suggest the presence of a clinical subtype characterized by a predominance of negative symptoms and cognitive impairment.

## Key Points

**Question:**

Does a distinct subtype of first-episode psychosis (FEP) characterized by predominant negative symptoms (negative symptom–dominant [NSD] psychosis) exhibit specific cognitive impairments?

**Findings:**

In this cross-sectional study, 788 patients with NSD exhibited more pronounced cognitive impairment compared with those with positive symptom–dominant or general symptom–dominant psychosis, particularly in processing speed and attention domains. The severity of cognitive impairment was positively associated with the intensity of negative symptoms, underscoring the pivotal role of negative symptoms in shaping cognitive deficits among individuals with first-episode psychosis.

**Meaning:**

The findings of this study suggest the existence of a distinct clinical subtype, NSD, within FEP, characterized by pronounced cognitive impairment.

## Teaser

This cross-sectional study compares the cognitive characteristics of patients with negative symptom–dominant first-episode psychosis with those of patients diagnosed with positive symptom–dominant and general symptom–dominant first-episode psychosis.

## Introduction

Psychosis is a severe mental disorder characterized by detachment from reality and encompassing symptoms such as hallucinations, delusions, and disorganized thinking. Cognitive impairments are commonly observed throughout the developmental trajectory of psychosis,^1^ from the prodromal phase^2,3,4^ to the first episode and the terminal stages of the illness. Patients with psychosis manifest deficits in attention, memory, executive function, and social cognition.^5,6^ These cognitive deficits substantially impact daily activities and quality of life.^7^ In addition to cognitive impairment, psychosis is associated with positive (eg, hallucinations and delusions) and general (eg, depressive and anxious mood) symptoms. However, negative symptoms, such as diminished emotional expression, social withdrawal, decreased motivation, and reduced speech, are core features of psychosis, involving a reduction in or absence of normal behaviors, emotions, and motivations, and appear to be associated with cognitive functioning.^8^ Studies have consistently reported a robust association between negative symptoms and cognitive impairment,^9,10,11^ specifically in attention, memory, and executive functioning.^6^

In contrast, although cognitive deficits may occur in individuals with positive or general symptoms, the association between cognitive functioning and these symptoms is less evident than that between cognitive functioning and negative symptoms.^12^ This highlights the unique cognitive characteristics associated with negative symptoms of psychosis. Understanding the intricate association between cognitive function and the negative symptoms of psychosis is paramount, as it can provide insights into the underlying mechanisms and support the development of targeted interventions.^13^ By addressing cognitive impairments that contribute to negative symptoms, clinicians can potentially improve overall outcomes and enhance the quality of life of patients with psychosis. Consequently, further research in this area is essential to advance our knowledge and refine clinical interventions for individuals with negative symptoms of psychosis.^14^

In this study, our aim was to conduct a comprehensive analysis of the associations between cognitive functioning and psychosis subtypes, including negative symptom–dominant (NSD), positive symptom–dominant (PSD), and general symptom–dominant (GSD) first-episode psychosis (FEP), while also addressing the methodologic limitations of previous research in this field. Previous studies^15,16,17^ had small sample sizes, confounding issues of concomitant antipsychotic use, and participants with late-stage illness. By evaluating a larger sample size and carefully controlling for potential confounding factors, our study sought to provide a more robust understanding of the specific cognitive characteristics associated with NSD psychosis.

## Methods

### Study Design and Participants

The cross-sectional study was led by the Shanghai Mental Health Centre (SMHC), and all procedures involving the participants were approved by the research ethics committee of SMHC. The research ethics committees at the different sites approved the study. Written informed consent was obtained from all participants during recruitment. For participants younger than 18 years, informed consent was provided by both the participants and their parents. No financial compensation was provided. All procedures conducted in this study complied with the ethical standards of the relevant national and institutional committees on human experimentation and the 1975 Declaration of Helsinki, revised in 2008. We followed the Strengthening the Reporting of Observational Studies in Epidemiology (STROBE) reporting guideline for observational studies.

Male and female patients aged 12 to 35 years meeting the diagnostic criteria for psychotic disorders of the *Diagnostic and Statistical Manual of Mental Disorders, Fourth Edition, Text Revision*, were recruited from 10 psychiatric tertiary hospitals in China. Data were collected from the National Key R&D Program of the Ministry of Science and Technology of China between 2016 and 2021. Overall, 788 patients with FEP who completed the Chinese versions of the Positive and Negative Symptoms Scale (PANSS)^18^ and Measurement and Treatment Research to Improve Cognition in Schizophrenia Consensus Cognitive Battery (MCCB)^19,20,21^ assessments were included in the data analysis. None of the participants in this study had previously received psychotropic medications. Furthermore, none met the exclusion criterion of a history of substance abuse or dependence. The sample size for this study was determined based on available resources meeting specified criteria within the investigation’s timeframe, rather than through formal statistical calculations. While the sample size was not predetermined, it aligns with the study’s criteria and objectives, reflecting the available population within the specified timeframe. Among the 788 individuals with FEP, there were 719 (91.2%) cases of schizophrenia, 33 (4.2%) cases of acute and transient psychotic disorder, 20 (2.5%) cases of persistent delusional disorder, and 16 (2.0%) cases of schizoaffective disorder.

### Clinical Symptom Assessments

Clinical psychopathologic status was assessed using the PANSS,^18^ which consists of 30 items divided into 3 psychopathology subscales: positive (PANSS-P; items P1-P7), negative (PANSS-N; N1-N7), and general (PANSS-G; G1-G16). Each item (symptom) was rated on a 7-point Likert scale (1 = absent and 7 = extreme). Structured clinical interviews were conducted by 23 senior psychiatrists (T.Z., L.X., and Q.H.) who completed the training required for this type of investigation. The interrater reliability of the PANSS, as per the ratings of the trained interviewers, ranged from 0.76 to 0.92.

### Group Criteria

To make the 3 PANSS subscales comparable, we conducted a full sample-based *Z* score transformation. The NSD psychosis group was defined according to the *Z* scores of PANSS-N, which were higher than those of PANSS-P and PANSS-G. Similarly, the PSD psychosis group was defined according to the *Z* scores of PANSS-P, which were higher than those of PANSS-N and PANSS-G. The GSD psychosis group was defined according to the *Z* scores of PANSS-G, which were higher than those of PANSS-P and PANSS-N.

### Neurocognitive Function Assessments

Neurocognitive performance was assessed using the Chinese version of the MCCB.^19^ The MCCB assessment was performed following the standardized guidelines of the test manual, and consistent with the original MCCB, the following 8 subtests were included in the present study: (1) part A of the Trail Making test (Trail Making A), (2) symbol coding of the Brief Assessment of Cognition in Schizophrenia (BACS) (BACS symbol coding), (3) Category Fluency Test (Category Fluency), (4) Continuous Performance Test–Identical Pairs (CPT-IP), (5) spatial span of the Wechsler Memory Scale-III (WMS-3 spatial span), (6) Hopkins Verbal Learning Test–Revised (HVLT-R), (7) Brief Visuospatial Memory Test–Revised (BVMT-R), and (8) Neuropsychological Assessment Battery: Mazes (NAB mazes). The interrater reliability of the MCCB, as per the ratings of the 12 trained raters, ranged from 0.80 to 0.96.

To mitigate potential biases, we implemented measures to enhance the reliability and validity of data collection. The research team underwent comprehensive training in PANSS application to ensure consistency and reduce interrater variability. Additionally, rigorous protocols were enforced during neurocognitive assessments using the Chinese version of the MCCB in the development and validation of which our research team actively contributed.

### Statistical Analysis

Data analysis was conducted in 2023. The patients were divided into NSD, PSD, and GSD groups, and neurocognitive performance was compared. Quantitative variables are expressed as means (SDs) and qualitative variables as frequencies (percents). Analysis of variance was used to examine quantitative variables and the χ^2^ test was used to examine qualitative variables, with a 2-tailed, paired significance threshold of *P* < .05. For pairwise comparisons, post hoc least significant difference correction was applied to adjust for multiple testing. Effect sizes were calculated using Cohen *d* for mean comparisons. The mean scores of the neurocognitive tests in the 3 groups were converted to *Z* scores on the basis of the mean (SD) of the overall sample. The association between clinical symptoms and neurocognitive performance was explored via the Pearson correlation coefficient test, and the *P* value was corrected (*P* < .05 / 24 was considered significant) by controlling the familywise error at the 0.0021 level using Bonferroni correction. Given that there were 3 groups (NSD, PSD, and GSD) and 8 MCCB tests, a total of 24 correlation analyses were conducted. In addition, logistic regression was performed to examine the discriminatory power of individual neurocognitive variables in identifying patients with NSD while controlling for age and sex. In the logistic analysis, the NSD group was coded as 1 and the PSD and GSD groups were coded as 0. Statistical analysis was performed with SPSS Statistics for Windows, version 20.0 (IBM Corp).

## Results

### Demographic and Clinical Characteristics

In total, 788 participants (men, 399 [50.6%]; women, 389 [49.4%]) with a median age of 22 (IQR, 17-28) years were included. The demographic and clinical characteristics of the 788 participants are reported in Table 1. There were no significant differences in mean age among the NSD, PSD, and GSD groups (*F* = 1.707; *P* = .18). The proportion of men and women was equivalent among the groups (*χ^2^* = 0.243; *P* = .89).

**Table 1.** Demographic, Clinical, and Cognitive Characteristics and Comparisons Among NSD, PSD, and GSD Groups

Neurocognitive variable	Mean (SD)				Comparison among 3 groups	
	Overall	NSD	PSD	GSD	*F*/*χ^2^*	*P* value
Patients, No. (%)	788	260 (33.0)	307 (39.0)	221 (28.0)	NA	NA
Age, y	22.71 (6.262)	22.13 (6.092)	22.96 (6.241)	23.06 (6.465)	1.707	.18
Sex, No. (%)						
Male	399 (50.6)	130 (50.0)	154 (50.2)	115 (52.0)	0.243	.89
Female	389 (49.4)	130 (50.0)	153 (49.8)	106 (48.0)		
Education, y	11.60 (3.053)	11.44 (3.013)	11.65 (3.071)	11.73 (3.079)	0.607	.55
Father education	9.68 (3.546)	9.56 (3.094)	10.15 (3.669)	9.24 (3.700)	2.891	.06
Mother education	8.71 (3.732)	8.74 (3.501)	9.12 (3.862)	8.23 (3.736)	2.366	.10
Family history of mental illness, No. (%)^a^						
None	702 (89.1)	227 (87.3)	275 (89.6)	200 (90.5)	1915	.75
Low risk of psychosis	56 (7.1)	20 (7.7)	22 (7.2)	14 (6.3)		
High risk of psychosis	30 (3.8)	13 (5.0)	10 (3.2)	7 (3.2)		
Symptom type						
Positive	21.54 (6.061)	18.30 (5.773)	25.29 (5.169)	20.11 (4.582)	136.929	<.001
Negative	18.13 (7.268)	23.02 (6.867)	14.37 (5.571)	17.60 (6.478)	134.870	<.001
General	39.72 (8.401)	37.70 (8.358)	37.16 (7.028)	45.64 (7.234)	95.027	<.001
PANSS total score	79.35 (16.891)	78.93 (18.254)	76.81 (15.631)	83.35 (16.235)	9.968	<.001
Trail Making A	49.78 (30.375)	54.34 (34.013)	47.47 (24.342)	47.62 (32.831)	4.410	.01
BACS symbol coding	46.46 (12.367)	44.60 (12.842)	47.84 (12.260)	46.73 (11.710)	4.957	.007
HVLT-R	20.58 (6.162)	19.79 (6.295)	21.02 (5.931)	20.87 (6.260)	3.198	.04
WMS-3 spatial span	14.26 (3.629)	13.93 (3.624)	14.38 (3.582)	14.48 (3.686)	1.671	.19
NAB mazes	12.26 (7.006)	11.60 (6.995)	12.39 (6.945)	12.84 (7.072)	1.974	.14
BVMT-R	20.72 (8.234)	19.98 (8.593)	20.95 (7.638)	21.26 (8.573)	1.635	.20
Category Fluency	17.89 (5.671)	17.25 (5.942)	18.08 (5.223)	18.37 (5.894)	2.627	.07
CPT-IP	0.83 (0.031)	1.73 (0.833)	1.87 (0.810)	1.91 (0.850)	3.057	.05

Abbreviations: BACS, Brief Assessment of Cognition in Schizophrenia symbol coding; BVMT-R, Brief Visuospatial Memory Test–Revised; CPT-IP, Continuous Performance Test–Identical Pairs; GSD, general symptom dominant; HVLT-R, Hopkins Verbal Learning Test–Revised; NA, not applicable; NAB, Neuropsychological Assessment Battery mazes; NSD, negative symptom dominant; PANSS, Positive and Negative Symptoms Scale PSD, positive symptom dominant; WMS-3, Wechsler Memory Scale-III spatial span.

^a^ None: no family members with mental disorders; low risk, a first-degree relative with nonpsychotic disorders; high risk, at least 1 first-degree relative with psychosis.

### Neurocognitive Function Profile and Comparisons

**Figure 1.**
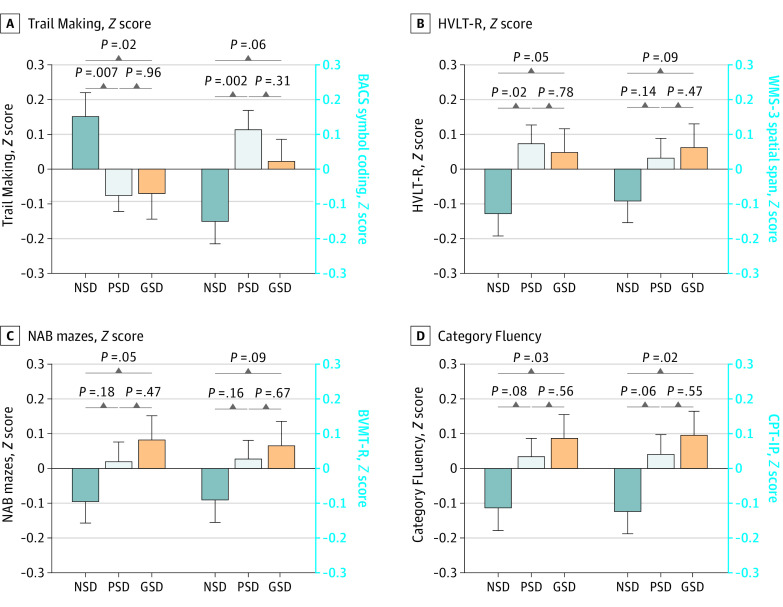
Comparisons of Neurocognitive Performance Among Patients With Negative Symptom Dominant (NSD), Positive Symptom Dominant (PSD), and General Symptom Dominant (GSD) First-Episode Psychosis BACS indicates Brief Assessment of Cognition in Schizophrenia; BVMT-R, Brief Visuospatial Memory Test–Revised; CPT-IP, Continuous Performance Test–Identical Pairs; HVLT-R, Hopkins Verbal Learning Test–Revised; NAB, Neuropsychological Assessment Battery: Mazes; and WMS-3, Wechsler Memory Scale-III spatial span. Whiskers indicate the SD.

Mean (SD) results of Trail Making A (49.78 [30.375]) were significantly higher (*F* = 4.410; *P* = .01) and those of BACS symbol coding (46.46 [12.367]; (*F* = 4.957; *P* = .007), HVLT-R (20.58 [6.162]; *F* = 3.198; *P* = .04), and CPT-IP (0.83 [0.031]; *F* = 3.057; *P* = .05) were significantly lower in the NSD than in the PSD and GSD groups (Table 1). Figure 1 shows the pairwise comparison results. Patients in the NSD group performed at a lower level than those in the PSD (mean difference = 6.866; SE = 2.549; *P* = .007) and GSD (mean difference = 6.719; SE = 2.549; *P* = .02) groups on the Trail Making A test. Patients in the PSD group performed better on the BACS symbol coding (mean difference = −3.241; SE = 1.037; *P* = .002) and HVLT-R (mean difference = −1.236; SE = 0.519; *P* = .02) tests than did those in the NSD group, whereas those in the GSD group performed better on the Category Fluency (mean difference = −1.123; SE = 0.519; *P* = .03) and CPT-IP (mean difference = 0.181; SE = −0.078; *P* = .02) tests.

### Effect Sizes

**Figure 2.**
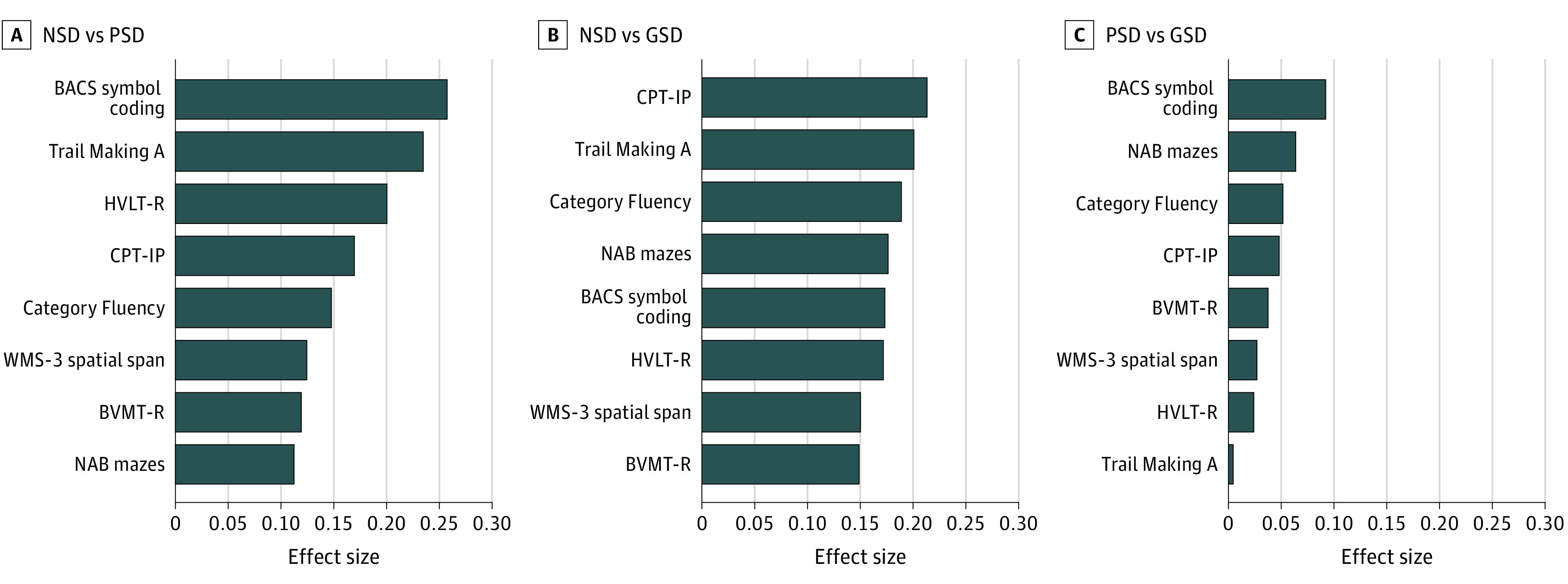
Effect Sizes (Cohen *d*) for Comparisons Between Negative Symptom Dominant (NSD), Positive Symptom Dominant (PSD), and General Symptom Dominant (GSD) Groups BACS indicates Brief Assessment of Cognition in Schizophrenia symbol coding; BVMT-R, Brief Visuospatial Memory Test–Revised; CPT-IP, Continuous Performance Test–Identical Pairs; ; GSD: general symptom dominant; HVLT-R, Hopkins Verbal Learning Test–Revised; NAB, Neuropsychological Assessment Battery mazes; NSD: negative symptom dominant; PSD: positive symptom dominant; WMS-3, Wechsler Memory Scale–III spatial span.

Figure 2 presents the ESs across the 8 neurocognitive tests for comparison between the NSD, PSD, and GSD groups. In the comparison between the NSD and PSD groups, BACS symbol coding (effect size = 0.26), Trail Making A (effect size = 0.24), and HVLT-R (effect size = 0.20)—the top 3 tests—had the maximum ES. In the comparison between the NSD and GSD groups, CPT-IP (effect size = 0.21), Trail Making A (effect size = 0.20), and BACS Category Fluency (effect size = 0.19)—the top 3 tests—had the maximum ES. The ESs for comparisons between the PSD and GSD groups were smaller than those between the NSD and PSD as well as the NSD and GSD groups.

### Correlations

**Figure 3.**
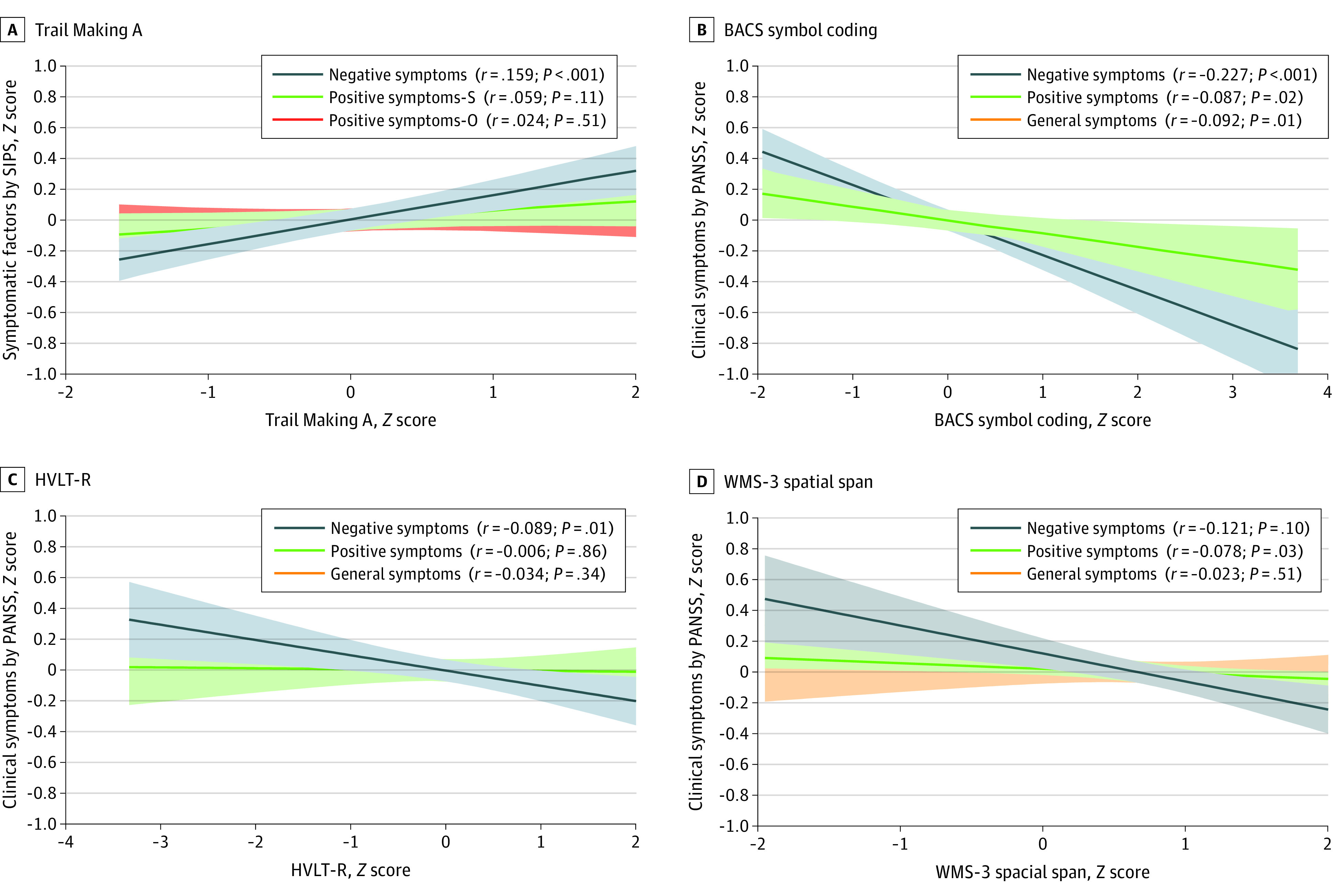
Correlations Between Clinical Symptoms and Neurocognitive Performances Using Trail Making A, Brief Assessment of Cognition in Schizophrenia (BACS) symbol coding, Hopkins Verbal Learning Test–Revised (HVLT-R), and Wechsler Memory Scale-III (WMS-3) Spatial Span Tests Corrected *P* values by controlling the familywise error at the .0021 (*P* < .05 / 24 was significant) level, using a Bonferroni correction. Shading indicates 95% CIs.

The scores for negative symptoms were associated with Trail Making A (*r* = 0.159; *P* < .001), BACS symbol coding (*r* = −0.227; *P* < .001), WMS-3 spatial span (*r* = −0.121; *P* = .001), NAB mazes (*r* = −0.119; *P* < .001), Category Fluency (*r* = −0.135; *P* < .001), and CPT-IP (*r* = −0.182; *P* < .001). Specifically, having a higher level of negative symptoms was associated with lower neurocognitive performance. However, an association was not observed between neurocognitive tests and positive and general symptoms, except for a correlation between NAB mazes and positive symptoms (*r* = −0.147; *P* < .001) (Figure 3; eFigure in Supplement 1).

### Discrimination

The standardized regression and raw regression coefficients are reported in Table 2. All neurocognitive tests, except the WMS-3 spatial span test (***χ^2^*** = 3.286; *P* = .07), significantly discriminated NSD, with significant differences observed across a range of tests, from BVMT-R (χ^2^ = 3.968; *P* = .05) to BACS symbol coding (χ^2^ = 9.765; *P* = .002). Age, but not sex, was a significant confounder in the Trail Making A (χ^2^ = 4.149; *P* = .04), BACS symbol coding (χ^2^ = 4.289; *P* = .04), WMS-3 spatial span (χ^2^ = 3.917; *P* = .048), NAB mazes (χ^2^ = 5.07; *P* = .02), and BVMT-R (χ^2^ = 3.867; *P* = .05) tests.

**Table 2.** Logistic Regression for Individual Neurocognitive Function Discrimination NSD From PSD and GSD, Adjusted by Age and Sex

Variable	β (SE)	OR (95% CI)	χ^2^ test	*P* value
Trail Making A	0.007 (0.002)	1.007 (1.003 to 1.012)	8.966	.003
Age	−0.025 (0.012)	0.975 (0.952 to 0.999)	4.149	.04
Sex				
Male	−0.076 (0.153)	0.927 (0.687 to 1.252)	0.244	.62
Female	NA	1.0 [Reference]	NA	
Constant	−0.475 (0.319)	0.622^a^	2.216	.14
BACS symbol coding	−0.020 (0.006)	0.981 (0.969 to 0.993)	9.765	.002
Age	−0.026 (0.012)	0.975 (0.951 to 0.999)	4.289	.04
Sex				
Male	−0.110 (0.154)	0.895 (0.662 to 1.211)	0.513	.47
Female	NA	1.0 [Reference]	NA	
Constant	0.828 (0.438)	2.289^a^	3.575	.06
HVLT-R	−0.032 (0.012)	0.969 (0.945 to 0.993)	6.431	.01
Age	−0.023 (0.012)	0.977 (0.954 to 1.001)	3.447	.06
Sex				
Male	−0.086 (0.154)	0.918 (0.678 to 1.242)	0.310	.58
Female	NA	1.0 [Reference]	NA	
Constant	0.490 (0.402)	1.633^a^	1.490	.22
WMS-3 spatial span	−0.038 (0.021)	0.963 (0.924 to 1.003)	3.286	.07
Age	−0.024 (0.012)	0.976 (0.952 to 1.000)	3.917	.05
Sex				
Male	−0.036 (0.153)	0.965 (0.714 to 1.303)	0.055	.81
Female	NA	1.0 [Reference]	NA	
Constant	0.395 (0.424)	1.485^a^	0.870	.35
NAB mazes	−0.025 (0.011)	0.975 (0.954 to 0.997)	4.855	.03
Age	−0.028 (0.013)	0.972 (0.948 to 0.996)	5.074	.02
Sex				
Male	0.004 (0.155)	1.004 (0.741 to 1.360)	0.001	.98
Female	NA	1.0 [Reference]	NA	
Constant	0.230 (0.349)	1.259^a^	0.435	.51
BVMT-R	−0.019 (0.009)	0.982 (0.964 to 1.000)	3.968	.05
Age	−0.024 (0.012)	0.976 (0.952 to 1.000)	3.867	.05
Sex				
Male	−0.100 (0.154)	0.905 (0.669 to 1.224)	0.420	.52
Female	NA	1.0 [Reference]	NA	
Constant	0.271 (0.381)	1.311^a^	0.507	.48
Category Fluency	−0.028 (0.014)	0.972 (0.947 to 0.999)	4.165	.04
Age	−0.020 (0.012)	0.980 (0.957 to 1.004)	2.655	.10
Sex				
Male	−0.053 (0.153)	0.949 (0.703 to 1.281)	0.118	.73
Female	NA	1.0 [Reference]	NA	
Constant	0.268 (0.371)	1.308^a^	0.523	.47
CPT-IP	−0.213 (0.095)	0.808 (0.671 to 0.974)	5.015	.03
Age	−0.022 (0.013)	0.978 (0.954 to 1.003)	3.021	.08
Sex				
Male	0.009 (0.157)	1.009 (0.742 to 1.373)	0.003	.95
Female	NA	1.0 [Reference]	NA	
Constant	0.195 (0.341)	1.215^a^	0.327	.57

Abbreviations: β, regression coefficient; BACS, Brief Assessment of Cognition in Schizophrenia symbol coding; BVMT-R, Brief Visuospatial Memory Test–Revised; CPT-IP, Continuous Performance Test–Identical Pairs; HVLT-R, Hopkins Verbal Learning Test–Revised; NA, not applicable; NAB, Neuropsychological Assessment Battery mazes; OR, odds ratio; WMS-3, Wechsler Memory Scale-III spatial span.

^a^ The constant OR does not have a 95% CI associated with it, as it represents the baseline value and is not subject to variation.

## Discussion

In this study, we conducted a large-scale investigation of Chinese patients with FEP and obtained several key findings. To our knowledge, this is the largest study to compare patients with FEP who are drug-naive with NSD psychosis with those with PSD and GSD psychosis in China. We found that patients with NSD exhibited more pronounced cognitive impairment than did those with PSD and GSD. Specifically, the cognitive differences between the NSD and PSD groups as well as the NSD and GSD groups were most notable in the domains of processing speed (BACS symbol coding, Trail Making A, and Category Fluency) and attention (CPT-IP). Patients with PSD and GSD showed no significant cognitive differences. Cognitive impairment was positively associated with the severity of negative symptoms. Most cognitive function tests used in our study were able to differentiate patients with NSD from those with PSD and GSD, which implies that cognitive assessments may serve as valuable tools for identifying and distinguishing patients with NSD.

In our study, patients with predominantly negative symptoms exhibited cognitive impairment, particularly in the domains of processing speed and attention, which may be related to the neural mechanisms associated with these symptoms. The negative symptoms of psychosis are thought to arise from disruptions in genetic variants of the dopamine pathway^22^ and hypodopaminergic activity in the mesocortical pathway and prefrontal cortex,^23^ which are responsible for cognitive and emotional processing. The dysfunction of these pathways may lead to slower information processing and lower attention, resulting in deficits in cognitive performance. Moreover, negative symptoms concerning the prefrontal and anterior cingulate cortices are often associated with structural and functional abnormalities in certain brain regions.^24^ A recent meta-analysis^24^ of structural and functional brain abnormalities in patients with schizophrenia and persistent negative symptoms suggested that structural brain abnormalities are consistently located in the prefrontal, temporal, limbic, and subcortical regions, and functional alterations are concentrated in the thalamocortical circuits and the default mode network. These neurobiological abnormalities may directly contribute to the cognitive impairments observed.^25,26,27^

Additionally, negative symptoms, such as reduced motivation and social withdrawal, may contribute to decreased cognitive engagement and practice in daily activities, leading to a decline in cognitive abilities over time. Lack of engagement in cognitive tasks and reduced environmental stimulation may further exacerbate processing speed and attention impairments in these patients.^28^ Dominant negative symptoms can also decrease the ability of patients to focus on tasks or sustain attention for extended periods. An emotional antisaccade task from Navalón et al^29^ found hypersensitivity to threat in patients with PSD schizophrenia and desensitization to socioemotional information in patients with NSD. Lack of motivation may result in reduced cognitive effort and attentional resources allocated to tasks, leading to cognitive impairment.

The association between negative symptoms and cognitive impairment is likely bidirectional. While negative symptoms may contribute to cognitive deficits, cognitive impairment can also influence the severity and manifestation of these symptoms.^30,31^ For example, reduced processing speed and attention may hinder social and occupational functioning, leading to increased social withdrawal and diminished motivation. Further research is needed to investigate the precise mechanisms underlying the associations between negative symptoms, processing speed, and attentional impairment. Longitudinal studies could help elucidate the temporal association between these variables and provide insights into whether cognitive impairment precedes or follows the onset of negative symptoms.

Our findings highlight the existence of a clinical subtype within the population of patients with psychosis that is characterized by a predominance of negative symptoms and cognitive impairment.^32,33^ This subtype represents a distinct subgroup with unique clinical features that warrant further investigation and consideration in clinical practice.^32^ Identification of this specific clinical subtype has important implications for understanding the heterogeneity of schizophrenia and tailoring treatment approaches.^34,35^ Traditional diagnostic criteria for schizophrenia primarily focus on positive symptoms, such as hallucinations and delusions. However, our study highlights the need to recognize and address prominent negative symptoms and cognitive impairment experienced by some patients. Interventions that combine pharmacologic approaches with psychosocial interventions,^36^ such as cognitive remediation^37^ and social skills training,^38^ may be particularly beneficial for individuals with this subtype.

These findings underscore the importance of considering premorbid neurodevelopmental disorders, such as autism spectrum disorder, in the assessment of individuals with FEP, particularly those presenting with predominantly negative symptoms. Cognitive deficits observed in patients with FEP with predominant negative symptoms may overlap with those seen in individuals with autism spectrum disorder, including difficulties in attention, executive function, and processing speed. Therefore, it is crucial for clinicians to conduct a comprehensive assessment, including a detailed developmental history, to differentiate between FEP and premorbid neurodevelopmental disorders. Additionally, interventions tailored to address specific cognitive deficits and social impairments associated with autism spectrum disorder may be warranted, particularly in individuals presenting with negative symptoms in the absence of hallucinations or delusions.

### Limitations

Our study had several limitations. First, the use of a cross-sectional design restricted our ability to understand temporal changes in negative symptoms and cognitive functions. Furthermore, the assessment of negative symptoms primarily relied on observer-rated measures, which may have introduced bias and variability. Observer-rated assessments can be influenced by individual perceptions and the subjectivity of the raters. Including more objective measures, such as digital biomarkers,^39^ would enhance the validity and reliability of negative symptom assessment. Considering that cognitive impairments observed in patients with negative symptoms may be influenced by their IQ, another limitation is the lack of IQ measurements in our study. Including IQ assessment would provide a better understanding of cognitive functioning and allow for a more comprehensive analysis of cognitive impairment concerning negative symptoms. Additionally, we did not consider the influence of the duration of untreated psychosis on our findings. Duration of untreated psychosis has been shown to be associated with various clinical outcomes,^40,41^ including symptom severity and cognitive functioning. The inability to account for duration of untreated psychosis in our study limited our understanding of this important variable on the association between negative symptoms and cognitive impairment.

## Conclusions

This cross-sectional study provides findings suggesting that patients with FEP with NSD exhibit more-pronounced cognitive impairments than do those with PSD and GSD, particularly in the domains of processing speed and attention. The observed cognitive deficits in processing speed and attention in patients with NSD suggest that they may experience difficulties in efficiently processing information and maintaining sustained attention, which has major implications for daily functioning, social interactions, and overall quality of life. Our findings underscore the need for a personalized approach to assessing and managing cognitive deficits associated with negative symptoms, which can potentially improve functional outcomes and enhance the overall well-being of patients with FEP.

## Notes

Supplement 1. **eFigure**. Correlations Between Clinical Symptoms and Neurocognitive Performances

Supplement 2.
Data Sharing Statement
