# Supplementary material for: Cognitive Impairments in Drug-Naive Patients With First-Episode Negative Symptom–Dominant Psychosis
Source: JAMA Netw Open. 2024 Jun 6;7(6):e2415110. doi: 10.1001/jamanetworkopen.2024.15110 (PMC11157355; doi:10.1001/jamanetworkopen.2024.15110)
Supplement: Supplement 1. — eFigure. Correlations Between Clinical Symptoms and Neurocognitive Performances [file jamanetwopen-e2415110-s001.pdf]

Supplementary Online Content

Zhang T, Wei Y, Tang X, et al. Cognitive impairments in drug-naive patients with first-episode negative symptom–dominant psychosis. *JAMA Netw Open*. 2024;7(6):e2415110. doi:10.1001/jamanetworkopen.2024.15110

eFigure. Correlations Between Clinical Symptoms and Neurocognitive Performances

This supplementary material has been provided by the authors to give readers additional information about their work.

© 2024 Zhang T et al. *JAMA Network Open.*

Figure. Correlations between clinical symptoms and neurocognitive performances (NAB mazes, BVMT-R, Category Fluency, CPT-IP)

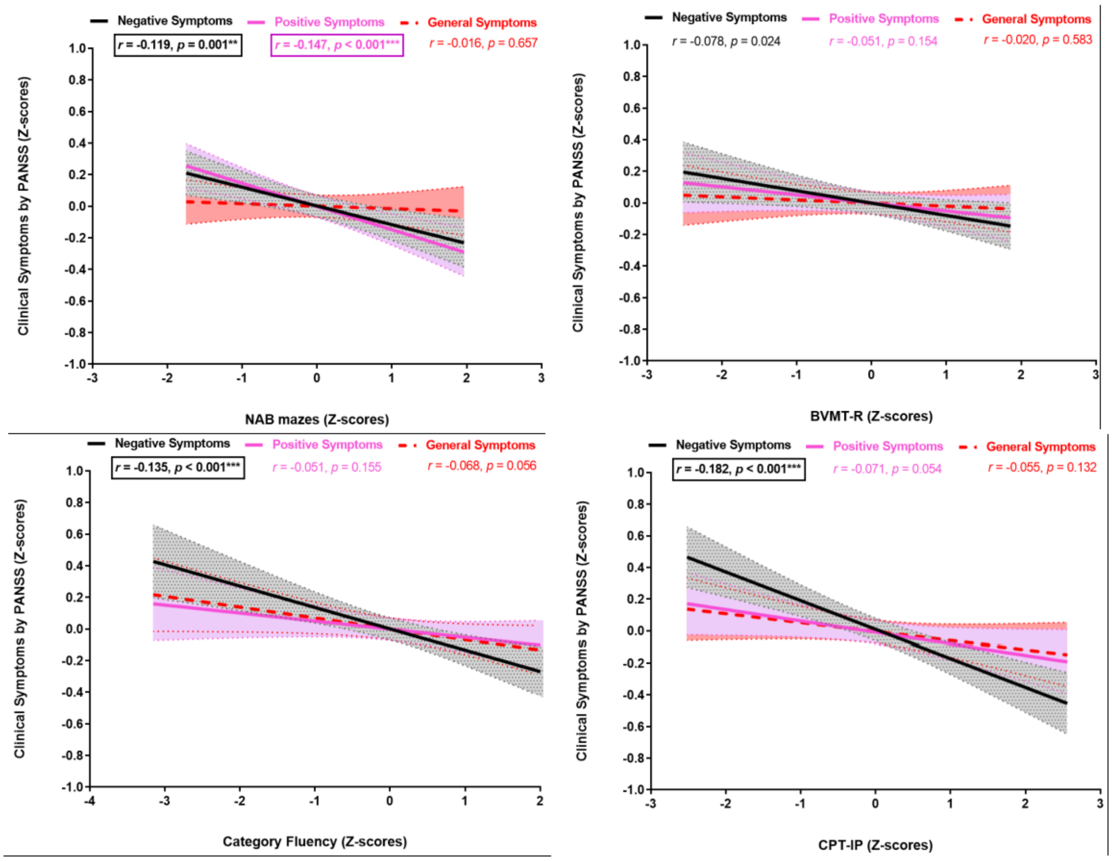

Notes: Corrected *p* by controlling the family-wise error, at the 0.0021 ($p < 0.05/24$ was significant) level using a Bonferroni correction. Abbreviations: BVMT-R, Brief Visuospatial Memory Test–Revised; CPT-IP, Continuous Performance Test–Identical Pairs; NAB, Neuropsychological Assessment Battery mazes.

© 2024 Zhang T et al. *JAMA Network Open.*
