# Supplementary material for: Cognitive Impairments in Drug-Naive Patients With First-Episode Negative Symptom–Dominant Psychosis
Source: JAMA Netw Open. 2024 Jun 6;7(6):e2415110. doi: 10.1001/jamanetworkopen.2024.15110 (PMC11157355; doi:10.1001/jamanetworkopen.2024.15110)
Supplement: Supplement 2. — Data Sharing Statement [file jamanetwopen-e2415110-s002.pdf]

Data Sharing Statement

Zhang. Cognitive Impairments in Drug-Naive Patients With First-Episode Negative Symptom–Dominant Psychosis. *JAMA Netw Open*. Published June 06, 2024.
doi:10.1001/jamanetworkopen.2024.15110

Data
Data available: Yes
Data types: Deidentified participant data
How to access data: Data will be available based on reasonable request from Dr. Tianhong Zhang(email: zhang_tianhong@126.com)
When available: With publication

Supporting Documents
Document types: None

Additional Information
Who can access the data: researchers whose proposed use of the data has been approved
Types of analyses: For a specified purpose, such as Meta-analysis.
Mechanisms of data availability: After approval of a proposal
